# Centromere Protein F (*CENPF*) Serves as a Potential Prognostic Biomarker and Target for Human Hepatocellular Carcinoma

**DOI:** 10.7150/jca.52187

**Published:** 2021-03-15

**Authors:** Yugang Huang, Xiuwen Chen, Li Wang, Tieyan Wang, Xianbin Tang, Xiaomin Su

**Affiliations:** 1 Department of Pathology, Taihe Hospital, Hubei University of Medicine, Hubei 44200, China.; 2 Department of Immunology, Nankai University School of Medicine, Tianjin 300110, China.

**Keywords:** *CENPF*, HCC, hepatocellular carcinoma, biomarker, survival, prognostic value, bioinformatics analysis.

## Abstract

Overexpression of Centromere Protein F (*CENPF*) is associated with tumorigenesis of many human malignant tumors. But the molecular mechanism and prognostic value of *CENPF* in patients with hepatocellular carcinoma (HCC) are still unclear. In this essay, expression of *CENPF* in HCC tumors were evaluated in a series of databases, including GEO, TCGA, Oncomine, GEPIA, The Human Protein Atlas and Kaplan-Meier plotter. It was apparent that mRNA and protein expression levels of *CENPF* were significantly increased in patients with HCC and were manifestly associated with the tumor stage of HCC. Aberrant expressions of CENPF were significantly linked with worse overall survival (OS) and progression-free survival (PFS) in HCC patients. Then, immunohistochemistry of* CENPF* in human HCC samples was carried out to suggest that CENPF protein was over-expressed in HCC tissues, compared with paired adjacent non-cancerous samples. And small interfering RNAs of *CENPF* in the human HepG2 cells were further performed to reveal that down-regulation of *CENPF* significantly inhibited cell proliferation, cell migration, and cell invasion, but slightly promoted cell apoptosis in human HepG2 cells. Moreover, the gene-set enrichment analysis (GSEA) was conducted to probe the biology process and molecular signaling pathway of *CENPF* in HCC. The GSEA analysis pointed out that *CENPF* was principally enriched in cell cycle and closely related to *E2F1* and *CDK1* in the regulation of cell cycle, especially during G2/M transition of mitosis in HCC. Additionally, immune infiltration analysis by CIBERSORTx revealed that mutilpe immune cells, including T_reg_, etc., were significantly different in HCC samples with CENPF^high^, compared with CENPF^low^. These results collectively demonstrated that* CENPF* might serve as a potential prognostic biomarker and novel therapeutic target for HCC. However, further research is needed to validate our findings and promote the clinical application of *CENPF* in HCC.

## Introduction

Hepatocellular carcinoma (HCC), as the most common primary liver neoplasms, is one of the most malignant tumors with high morbidity and mortality, which makes it a notable healthcare issue for human beings in the global world 1, 2. Liver neoplasms are the fourth leading cause of cancer-related death and ranks sixth among new cases worldwide 3, 4. Surpassing breast, prostate, and colorectal cancers, liver neoplasms is predicted to be the third leading cause of cancer-related death in Europe and the United States by 2030 5, 6. The World Health Organization estimates that the global incidence of HCC is rising and might reach one million cases annually in the next decade 7, 8. HCC has high molecular heterogeneity, with a poor prognosis 9, 10. Due to the lack of effective biomarkers to detect diseases and predict individual differences in patients, the mortality rate of HCC is high. Over 80% of patients are diagnosed with advanced liver cancer 11, 12. As the 5-year survival rate is only 18%, liver cancer is the second most fatal tumor after pancreatic cancer 13-15. For patients in Asian countries such as China, the situation is even more severe, with a 5-year survival rate reported as low as 12% 16, 17. So far, some biomarkers with potential diagnostic, prognostic or therapeutic value for HCC have been reported. Molecular studies 18-20 have shown that the most common variations in HCC include mutations in the *TERT* promoter, *TP53* and *CTNNB1*, copy number variation, abnormal DNA methylation 21, overexpression of *PD-L1* 22-24, etc 25. Although some of the above-mentioned biomarkers of HCC have aroused extensive concern, most of them were studied separately rather than as a part of the whole carcinogenesis process; the related studies are still in the preliminary investigation or clinical verification stage. Therefore, it is urgent to find reliable biomarkers to predict the early or accurate prognosis, and to develop new molecular targeted therapy strategies for HCC.

As a cell cycle-related nuclear antigen, the Centromeric protein F (*CENPF*) is expressed at low levels in G0/G1 cells and accumulates in the nuclear matrix in the S-phase, with the highest expression level in G2/M cells 26. The abnormal expression or activation of *CENPF* has been reported in several human malignant tumors, including HCC 27, 28, breast cancer 29, and other tumors 30. Additionally, elevated *CENPF* expression contributes to unregulated cell proliferation in HCC 28. Recent studies have shown that* CENPF* and *FOXM1* were important regulators of prostate cancer malignancies and prognostic indicators for poor survival and extensive tumor metastasis 31. Further studies have shown that* COUP* transcription factor 2 promoted prostate cancer metastasis through *CENPF* signal transduction 32. Generally, *CENPF* might emerge as a promising biomarker for predicting the prognosis of HCC. Therefore, identifying the masked mechanism of *CENPF*-mediated oncogenes or tumor suppressor genes as predictive biomarkers might provide new treatment strategies. Nevertheless, the divergences in expression levels, genetic alterations, biological functions and process, molecular mechanisms, and prognostic value of *CENPF* in HCC have not been fully expounded.

The advancement and development of gene microarray and RNA-sequencing technology has innovated the research of RNA and DNA, which has become an important method for biological and medical research 33-35. Based on the GEO, Oncomine and TCGA databases, this study expanded the relevant knowledge of HCC and comprehensively analyzed the relationship between *CENPF* and the pathogenesis and progression of HCC, in order to provide useful enlightenment for the occurrence and aggressiveness of HCC.

## Materials and Methods

### Data resource and description

As a publicly available genomics database, Gene Expression Omnibus (GEO) of NCBI was systematic and complete queried for all datasets related to studies of HCC, which collects submitted high-throughput gene expression data worldwide. Following criteria were considered qualified for our analysis: (1) The research objects included human HCC and its adjacent or normal liver tissue. (2) The information of technology and platform used for studies was detailed. (3) The number of samples was >10. Based on these criteria, eight gene expression microarray datasets for HCC, including GSE14520, GSE60502, GSE40367, GSE84005, GSE112791, GSE76297, GSE25097, and GSE87630, were downloaded from the repository.

HCC mRNA normalized counts data of TCGA, derived from RNA-seq Htseq platform, were downloaded from Genomic Data Commons (GDC) Data Portal. TCGA RNA-seq data contains 424 samples, including 374 primary HCC tumor and 50 normal liver samples. RTCGA Toolbox 36 and edgeR 37 packages were applied to detect the expression of *CENPF* in HCC and normal tissues.

### Identification *CENPF* expression by Bioinformatics strategy

As the currently world's largest oncogene chip database and integrated data mining platform, Oncomine is widely used to excavate cancer gene information. So far, the database has included gene expression data from 715 datasets and about 90,000 cancer and normal tissue samples, which can be used to explore the expression of *CENPF* in HCC and its related normal tissues. 38. Studies of *CENPF* expression in HCC and normal liver samples were selected with the thresholds as follows:* P*-value< 0.05 and the data type was restricted to mRNA expression levels in Oncomine database and visualized by GraphPad Prism. Moreover, protein expression of CENPF in HCC tissues and their associated normal tissues was screened in The Human Protein Atlas, which is a human proteomics online service database and designed to map human proteomics information in cells, tissues, and organs by using various omics technologies, including antibody-based imaging, mass spectrometry-based proteomics, transcriptomics, and systems biology.

Gene Expression Profiling Interactive Analysis (GEPIA, http://gepia.cancer-pku.cn/) is an interactive online database for detecting the mRNA expression of 9736 tumors and 8587 normal samples from the TCGA and Genotype-tissue Expression dataset (GTEx) projects 39. GEPIA can be performed for gene differential expression analysis, profiling plotting according to cancer types or pathological stages, correlation analysis, patient survival analysis, and similar gene analysis. The mRNA expression of *CENPF* in different stages or grades was compared between HCC and normal tissues by using the GEPIA dataset and TCGA RNA-seq data.

### Correlation between *CENPF* and clinicopathological characteristics in HCC

Based on HCC information from TCGA datasets, correlation between *CENPF* and clinic-pathological characteristics was investigated. The expression matrix from TCGA datasets contains 374 HCC tissues divided to two groups, including187 HCC samples with *CENPF* low expression (CENPF^low^) and 187 HCC samples with *CENPF* high expression (CENPF^high^) based on the median expression level of *CENPF* (median cutoffs). Grouping of risk types was conducted via 'ggrisk' package of R software (version 4.0.3) 40, 41. Sanguini diagram, which can be used to show the distribution trend of survival and expression of a gene in different stages, ages and other clinical characteristics for tumors, was built based on the 'ggalluval' package of R software 42.

Further, the univariate (uni-cox) and multivariate cox (mult-cox) regression analysis was performed to identify the proper terms to build the nomogram. The forest was used to show the *P-values*, HR and 95% confidence interval (CI) of each variable through 'forestplot' R package. A nomogram was developed based on the results of multivariate Cox proportional hazards analysis to predict the 1-year, 2-year, and 3-year overall recurrence 43. The nomogram provided a graphical representation of the factors, which can be used to calculate the risk of recurrence for an individual patient by the points associated with each risk factor through 'rms' R package 44.

### Survival analysis

Kaplan-Meier plotter is an online data service platform that contains microarray gene expression data and survival information from GEO, TCGA, and the Cancer Biomedical informatics Grid, which provides survival information of 374 HCC patients 45. In this study, the Kaplan-Meier plotter was conducted to evaluate the prognostic value of *CENPF* mRNA expression. The overall survival (OS), progression-free survival (PFS), 1-year, 3-year and 5-year OS of HCC patients were tested by dividing samples into two groups based on median expression (high expression and low expression) and assessed by using Kaplan-Meier survival plots, with a hazard ratio with 95% confidence intervals and log rank *P*-value. Further, subgroup survival analyses were conducted by dividing patients based on different population, pathological and histological subtypes.

### Gene-set enrichment analysis (GSEA) in HCC samples

In order to interpret the gene expression data between *CENPF* and other genes, and to identify the underlying pathways that correlate to HCC with CENPF^low^ or CENPF^high^, GSEA software (version 4.0.3, Broad Institute, USA) was performed, to probe the biological mechanisms based on TCGA datasets 46, 47. The predefined gene sets, including 'c2.cp.kegg.v7.2.symbols.gmt', 'c2.cp.biocarta.v7.2.symbols.gmt', and 'h.all. v7.2.symbols.gmt', from the Molecular Signatures Database were employed, respectively. A normalized enrichment score (NES) was calculated as the primary GSEA statistic. For the analysis results, the threshold values of statistical significance were set as |NES|> 1, normalized P-values (NOM P-values) < 0.05, and FDR < 0.25. Lastly, the results of the GSEA analysis were visualized via Sangerbox tools, a free online platform for data analysis (http://www.sangerbox.com/tool).

### Analysis of immune cell infiltration profile

Immune cell infiltration is an important index to predict immunotherapy 48. Based on TCGA dataset, analysis of immune cell infiltration was performed by CIBERSORTx, an analytical tool that provides an estimation of the abundances of member cell types in a mixed cell population by inputting normalized gene expression matrix 49. The results of the analysis present the abundance of 22 kinds of immune cells, including 7 types of T cells, 3 types of B cells, 2 types of natural killer (NK) cells, monocytes, 3 types of macrophages (Mφ), 2 types of dendritic cells (DC), 4 types of granulocytes (mast cells, eosinophils, and neutrophils). The expression matrix from TCGA dataset was normalized via 'Limma' package of R software, and all the results were visualized by applying the packages of R software. Moreover, the expression of five genes closely related to tumor immunotherapy, including *LAG3*, *CTLA4*, *HAVCR2*, *PD-1*, and* PD-L1* were further investigated in HCC by GEPIA 50.

### Analysis of the correlation between *CENPF* expression and MSI or TMB in HCC

As independent factors of immune response to PD-1 / PD-L1 mono-antibody, the microsatellite instability (MSI) and tumor mutation burden (TMB) have been clinically proven to play an outstanding role in predicting the anti-tumor effect of PD-1 / PD-L1 inhibitors 51, 52. The analysis of the correlation between* CENPF* expression and TMB/MSI in HCC based on TCGA were performed via 'ggstatsplot' package of R software. Spearman's correlation analysis was used to describe the correlation between quantitative variables without a normal distribution.* P-*value <0.05 was considered statistically significant. The range of correlation coefficient is (- 1, 1). A negative number represents a negative correlation between the two gene expressions, and a positive value represents a positive correlation. The closer the value is to 1 or - 1, the stronger the correlation between the two variables; the closer the value is to 0, the weaker the correlation between the two variables.

### Human HCC samples and immunohistochemistry

Screening criteria for PPFE (Formalin-fixed and paraffin embedded) samples of HCC patients were as follows: (1) All samples were stored at room temperature (20~25℃) and collected from patients who had been diagnosed as HCC from May 2019 to May 2020 at Taihe Hospital of Hubei University of Medicine, China. All HCC patients were diagnosed and graded according to the pathological characteristics by at least two pathologists in the Department of Pathology, Taihe Hospital, with a total of 81 cases. (2) Samples containing cancerous tissues and paired adjacent non-cancerous samples were screened out. Finally, 5 HCC tissues and 5 paired adjacent non-cancerous samples of PPFE were included. All human samples were obtained by informed consent (IFC) from patients or family members, and this study was supported and approved by the Ethics Committee of Taihe Hospital. Details of 5 enrolled patients with HCC were listed in Table S1.

Immunohistochemistry of paraffin embedded samples (IHC-P) was performed according to the manufacturer's recommended procedure. 3 μm of HCC tissue and para-cancerous tissue were taken from the PPFE. All sections were deparaffinized with xylene and rehydrated through a graded ethanol series. Endogenous peroxidase activity was blocked with 3% hydrogen peroxide in methanol for 10 min. Antigen retrieval was performed by EDTA at pH 9.0 in a pressure cooker for 4 min. After PBS washing (three times, 3 min each), slides were incubated with the rabbit anti-human CENPF polyclonal antibody (ab5, Abcam, 1/100 dilution) at 37℃ for 1 hour. After incubation with HRP labeled second antibody at 37℃ for 0.5 hour, nuclei were stained by hematoxylin for 30 seconds.

### Cell line and siRNA

Small interfering RNAs of *CENPF* (siCENPF) was designed to explore the role of CENPF in cell proliferation and migration of HCC in the hepatoma cell line, human HepG2. The qRT-PCR primers and small interfering RNAs of *CENPF* and reference gene were synthesized by Sangon Biotech (Shanghai, China), and the information of their sequences was listed in Table 1.

The small interfering RNA transfection was performed according to instructions of the manufacturer. Each group had at least 3 replicates.

### RNA isolation and quantitative real-time PCR

HepG2 cells after cells were transfected with 50 nmol of CENPF siRNA (siCENPF) or contorl siRNA (siNC) for 48 hours respectively. Total RNA was extracted from the HepG2 cells using TRIzol reagent (Invitrogen, USA). First-strand cDNA was generated from total RNA using oligo-dT primers and reverse transcriptase (Invitrogen, USA). Quantitative real-time PCR (qRT-PCR) was conducted using QuantiTect SYBR Green PCR Master Mix (Qiagen, Germany) and specific primers in an ABI Prism 7000 analyzer (Applied Biosystems, USA). GAPDH was detected in each experimental sample as an endogenous control. All the reactions were run in triplicate. The relative RNA levels of CENPF in HCC samples were calculated by using the 2^-ΔΔCt^ method.

### Western blotting

For western blotting, human HepG2 cells were transfected with 50 nmol of siCENPF) and siNC for 48 hours respectively, and then cells were collected. Cell lysates were denatured and subjected to SDS-PAGE, then were transferred to PVDF membranes (Millipore, USA). The membranes were incubated with primary antibody (CENPF antibody: ab5, Abcam, UK) overnight at 4℃. Membranes were washed for 4 times with washing buffer (three times TBST and at last one time TBS, 10 minutes each), and then incubated with the secondary HRP-conjugated antibody for 1.5 hour at room temperature. After washed by washing buffer for 4 times, the membranes were detected using an enhanced chemiluminescence assay with Lumi-Glo reagents (Millipore, USA).

### CCK-8 cell proliferation experiment

Human HepG2 cells were plated in 96-well plates at 100 μL (total 2×10^3^ cells), then transfected with 50 nmol of siCENPF and siNC respectively. 10 μL CCK-8 solution (Beyotime, China) was added to each well. The cells were incubated in the cell incubator for 0.5 hour and 1 hour. The absorbance (optical density, OD) representing cell density was measured at 450 nm.

### Wound healing assay

Cell migration was analyzed in wound healing assay. Human HepG2 cells were seeded in 12-well plates in DMEM with 10% fetal bovine serum (FBS), then cells were transfected with 50 nmol of siCENPF or siNC for 24 hours. Wounds were scratched by 20μl pipette tips. Each well was then rinsed 5 times with PBS to clear floating cells from scratches, and 3 mL of 10% FBS, 1% antibiotic-antimycotic DMEM was added to each well. Scratch regions were photographed at 0 and 24 hour.

### Transwell assay

Transwell assays were performed to analysis metastatic ability and invasion of the HepG2 cells. The metastatic ability of the cells was investigated by Transwell plates (Corning, USA). Cells were transfected with 50 nmol of siRNAs for 24 hours. Then Serum-free single cell suspensions were placed in the upper chamber per well. The lower chamber was filled with 500 uL 1640 medium with 20% FBS as a chemoattractant for 24 hours. For invasion assays, the membrane inserts were pre-coated with Matrigel. The cells were cultured for 24h. Cells in the lower surface of the membrane was fixed with 4% PFA and then stained with 0.5% crystal violet. Cells in ten random fields per chamber were counted and analyzed using Image J software. The percentage of migration was calculated and compared to the mock group.

### Flow cytometric analysis

Cells were transfected with 50 nmol of siRNAs for 24 hours. Then cells were analyzed using the Annexin V-FITC apoptosis detection kit (Vazyme Biotech, USA) as instrument. 1 ×10^4^ cells of each sample were counted in the flow cytometer (BD, Bangladesh).

### Statistical analysis

Statistical analysis was performed via GraphPad Prism (version 8.2.1, San Diego, CA) and SPSS 22.0 (IBM SPSS Inc. Chicago, IL) software. Student's* t-test* (two-tailed) were utilized for the comparison of two sample groups. Differences were considered as statistically significant when* P* < 0.05 (*P < 0.05, ** P < 0.01, *** P < 0.001, **** P < 0.0001).

## Results

### *CENPF* is overexpressed in HCC compared with normal liver tissues

**Figure 1:**
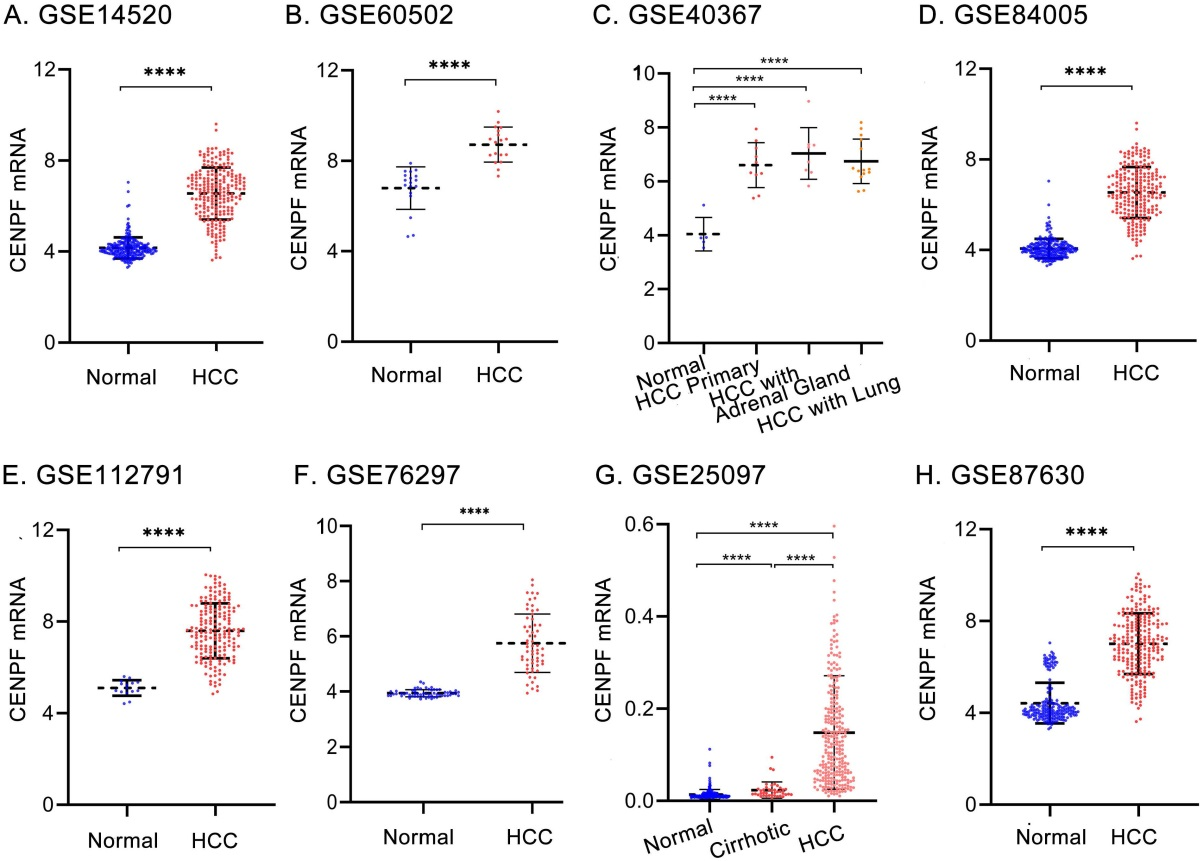
The expression of *CENPF* in HCC patients from GEO datasets. Relative mRNA expression levels of *CENPF* from (A) GSE14520, (B) GSE60502, (C) GSE40367, (D) GSE84005, (E) GSE112791, (F) GSE76297, (G) GSE25097, and (H) GSE87630 in normal livers and HCC samples. Pre-processed expression levels are Log_2_ normalized and median centered. Data were analyzed using un-paired student's t-test. Differences were viewed as statistically significant when *P* < 0.05. HCC: hepatocellular carcinoma. HCC with Adrenal Gland: HCC with adrenal gland metastasis. HCC with Lung: HCC with lung metastasis.

**Figure 2:**
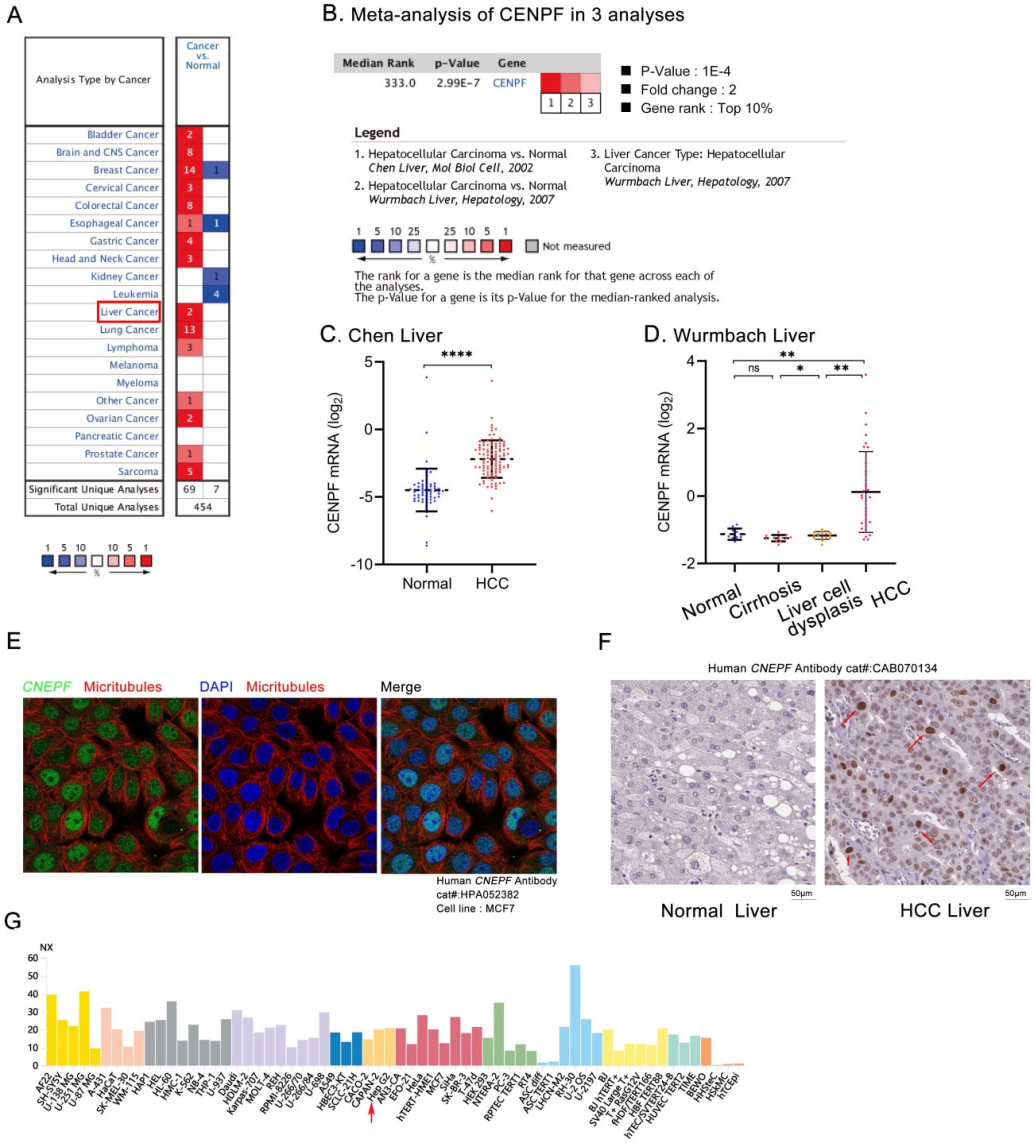
Comparison of *CENPF* expression in Oncomine and The Human Protein Atlas database. (A) The mRNA levels of *CENPF* in different types of cancers from Oncomine database. The graph shows the numbers of datasets with statistically significant mRNA over-expression (red) or down-regulated expression (blue) of *CENPF*. The threshold was designed as following parameters: *P*-value <0.05 and fold change ≥1.5; (B) Meta-analysis of *CENPF* expression in 3 analyses; (C) *CENPF* levels in normal, and HCC tissues in Chen Liver; (D) *CENPF* levels in normal, cirrhosis, liver cell dysplasia and HCC tissues in Wurmbach Liver; (E) Protein expression and localization of CENPF in MCF7 cell line by IFC; IFC: immunofluorescence. Green represents the protein CENPF, red represents microtubules, and blue represents nucleus. After merging, the protein CENPF is expressed in the nuclear region. (F) Protein expression of CENPF in normal, and HCC human tissues by IHC. The expression of CENPF was shown by the red arrow. IHC: immunohistochemistry. (G) The mRNA expression profile of *CENPF* in different human cell line, including HepG2 (shown by the red arrow).

**Figure 3:**
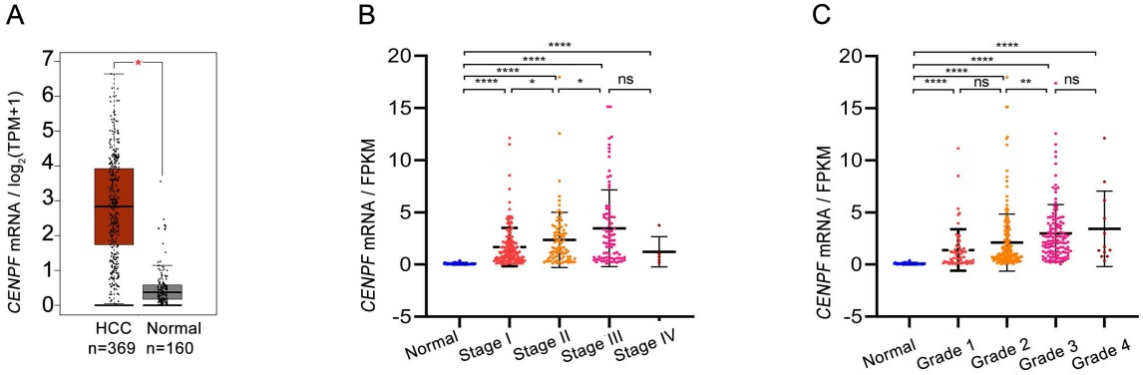
Correlation between the transcription levels of *CENPF* and the tumor stage/grade of patients with HCC. (A) The mRNA levels of *CENPF* in normal(n=160) and HCC tissues(n=369) from GEPIA. (B-C) The mRNA levels of *CENPF* in HCC in pTNM stage I-IV and grade 1-4 from TCGA database.

The details of GEO series involved in this study were presented in Table 2. As illustrated in Figure 1, *CENPF* mRNA was significantly overexpressed in GSE14520, GSE60502, GSE40367, GSE84005, GSE112791, GSE76297, GSE25097, and GSE87630 (all *P* < 0.01). For further validation, we performed meta-analysis of *CENPF* expression in Oncomine database. Compared with that in normal livers, *CENPF* was evidently upregulated in HCC tumors (*P* < 0.001, Figures 2A-D), but not in cirrhosis, and liver cell dysplasia (*P >* 0.05, Figure 2D). Moreover, study from The Human Protein Atlas declared that protein of CENPF is mainly expressed in the region of nucleoplasm by immunofluorescence (IFC) staining in MCF7 cells (Figure 2E). And CENPF was also high expressed in HCC patients (Figure 2F) or cell line, human HepG2 cells (Figure 2G), compared to normal ones in terms of protein or mRNA. The relationship between the transcription levels of *CENPF* and the tumor stage/grade in HCC patients were also analyzed by the GEPIA (Figure 3A) and TCGA (Figures 3B-C) dataset. As results, the mRNA expression level of *CENPF* was significantly and positively correlated with the tumor stage and grade for HCC.

### Correlation between *CENPF* and clinicopathological characteristics in patients with HCC

**Figure 4:**
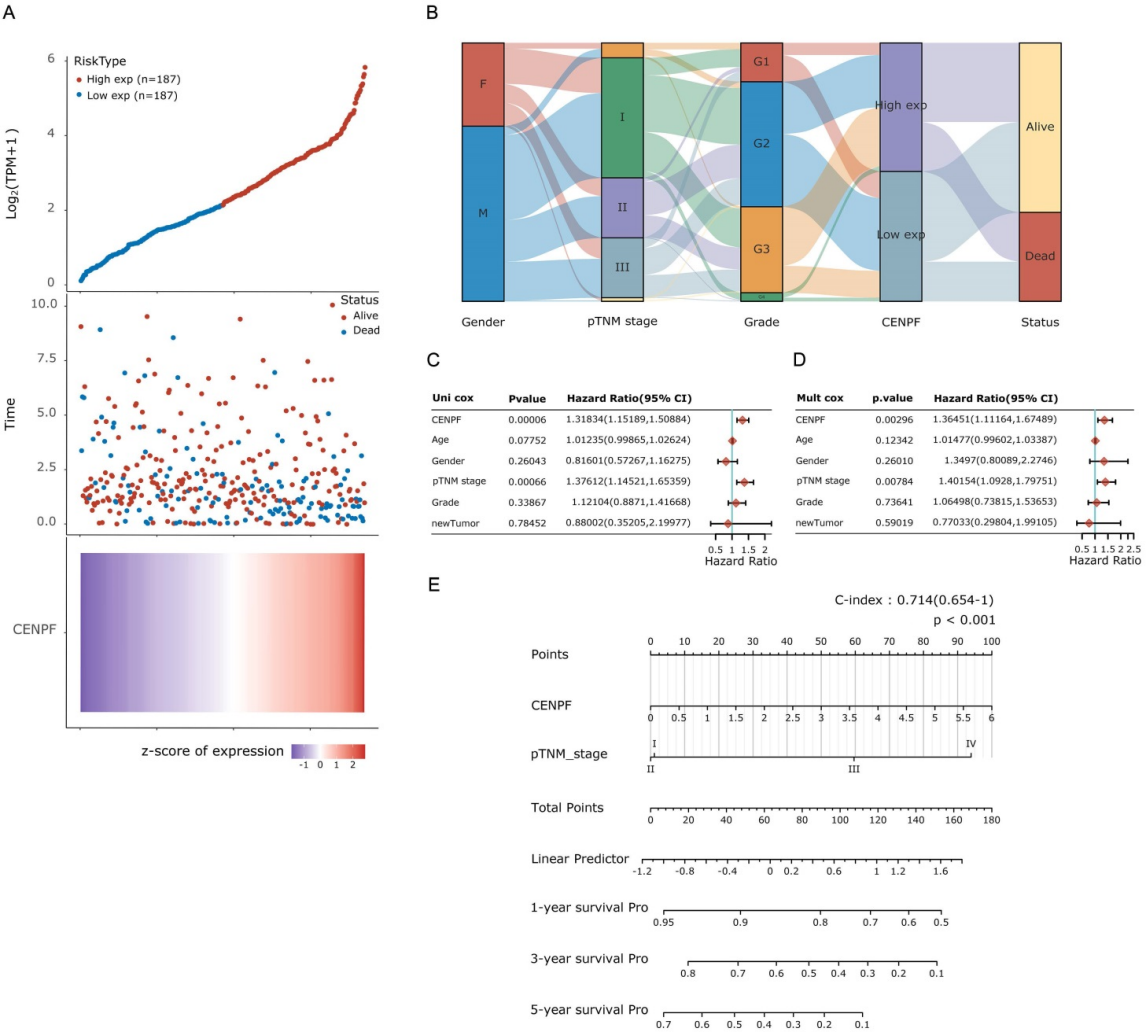
Correlation between the expression of *CENPF* and the prognosis in HCC based on TCGA datasets. (A) *CENPF* expression and survival status of HCC patients from TCGA datasets, in which the top represents the scatter plot of the gene expression from low to high, and is divided into two groups according to the median cutoffs; the middle represents the scatter plot distribution of survival time and survival status corresponding to gene expression of different samples; the bottom represents the heat map of the gene expression in the corresponding samples; the abscissa of the upper, middle and lower graphs in the graph represent the samples, and the sample order is consistent. (B) Sanguini diagram for outlining the distribution of CENPF expression in gender, pTNM stage and grade. Each column represents a characteristic variable, different colors represent different types or stages, and lines represent the distribution of the same sample in different characteristic variables. Hazard ratio and *P*-value of constituents involved in uni-cox (C) and mult-cox (D) regression and relevant clinical parameters of the *CENPF*. (E) Nomogram to predict 1-year, 2- year, and 3- year survival rate in HCC patients associated with *CENPF* expression and pTNM stage.

In order to explore the correlation between *CENPF* and clinicopathological characteristics in patients with HCC, all 374 HCC tissues were divided into two groups, including187 CENPF^low^ HCC samples and 187 CENPF^high^ HCC samples according to the median cutoffs of *CENPF* expression (Figure 4A). As listed in Table 3, BMI of all HCC patients were over 18.5 kg/m^2^, and more HCC cases had higher BMI in *CENPF* high group than those in *CENPF* low group (*P* = 0.025). Less HCC cases had family history of cancer in *CENPF* high group than those cases in *CENPF* low group (27.8% vs. 37.4%, *P* = 0.036). Incidence of new tumor events after initial treatment was higher in *CENPF* high group than those in *CENPF* low group (*P* = 0.038). However, HCC patients in *CENPF* low group suffered from less hepatic inflammation (39.6% vs 29.4, *P* = 0.029). Just as we expected, HCC patients with CENPF^high^ group experienced manifestly advanced neoplasm histologic grade (especially grade III, *P*<0.01) and advanced pathological stage (especially stage II and III, *P* = 0.032). Meanwhile, the distribution of *CENPF* expression in gender, pTNM stage and grade was shown in Figure 4B. Furthermore, the uni-cox and mult-cox regression analysis was performed to identify the effects of *CENPF* and clinical factors including age, gender, grade, pTNM stage, and new tumor events on the prognosis of HCC patients. According to the uni-cox analysis in Figure 4C, the CENPF expression and pTNM stage were correlated with the prognosis of HCC patients (all *P*<0.05). And according to the mult-cox analysis in Figure 4D, the CENPF expression and pTNM stage might be independent prognostic factors in HCC patients (all *P*<0.05). Based on uni-cox and mult-cox analysis, nomogram was constructed to predict 1-year, 2- year, and 3- year survival rate in one HCC patients associated with CENPF expression and pTNM stage (Figure 4E).

### Overexpression of *CENPF* predicts worse prognosis in HCC patients

**Figure 5:**
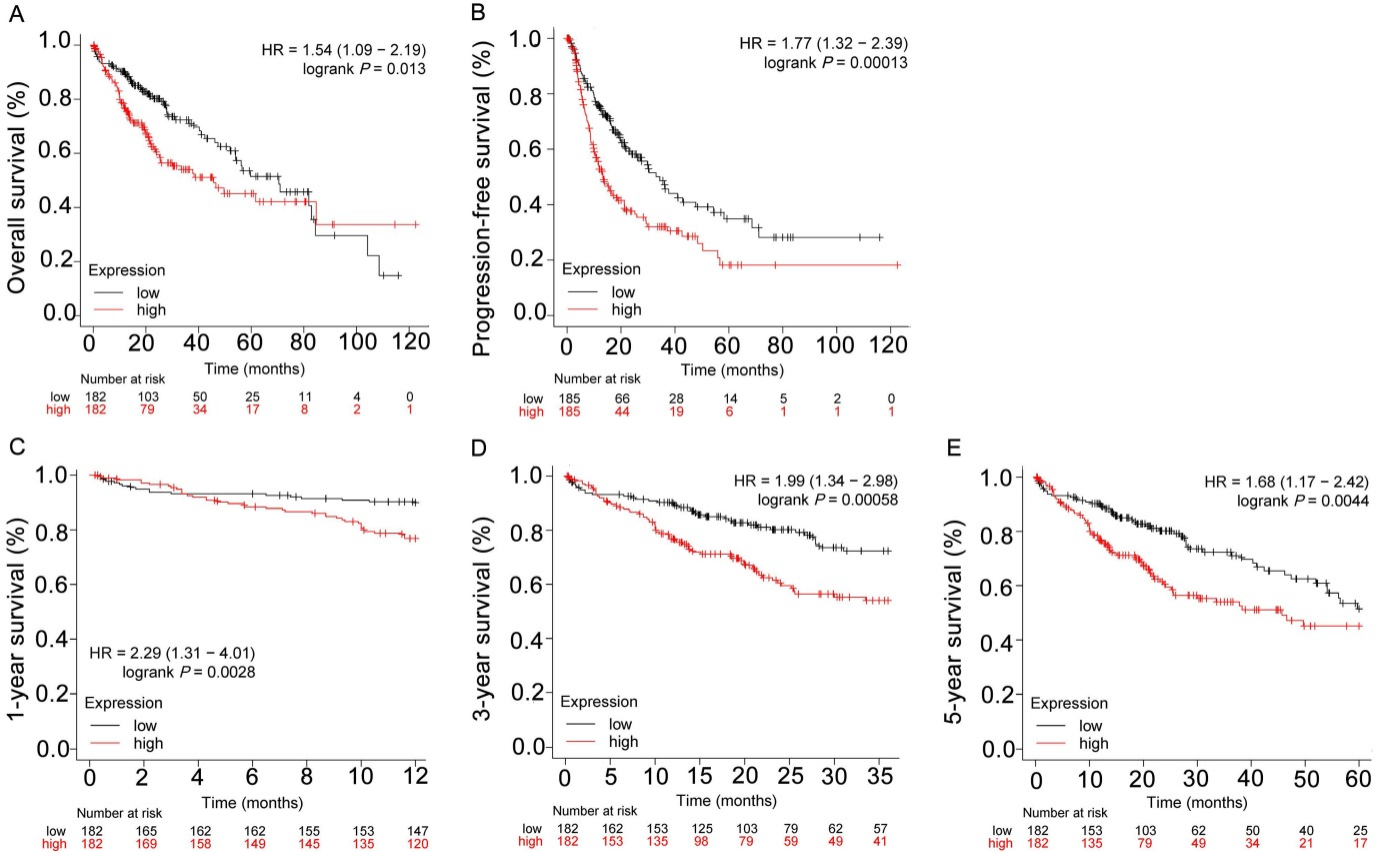
Overexpression of *CENPF* predicted worse prognosis in HCC patients (Kaplan-Meier plotter). The OS (A), PFS (B), 1-year (C), 3-year (D) and 5-year (E) survival curves comparing patients with high (red) and low (black) *CENPF* expression in HCC were plotted using Kaplan-Meier plotter database at the threshold of *P*-value < 0.05.

As validated in Kapan-Meier Plotter, overexpression of *CENPF* predicted worse OS (overall survival; HR = 1.54, log rank *P* = 0.013) and PFS (progression-free survival; HR = 1.77, log rank *P* = 0.00013) in HCC (Figures 5A-B). Meanwhile, subgroup analyses indicated that *CENPF* upregulation in tumors was a risk factor for 1-year, 3-year and 5-year OS in patients with HCC (HR = 2.29, log rank *P* = 0.0028; HR = 1.99, log rank *P* = 0.00058 and HR = 1.68, log rank *P* = 0.0044, respectively, Figures 5C-E).

**Figure 6:**
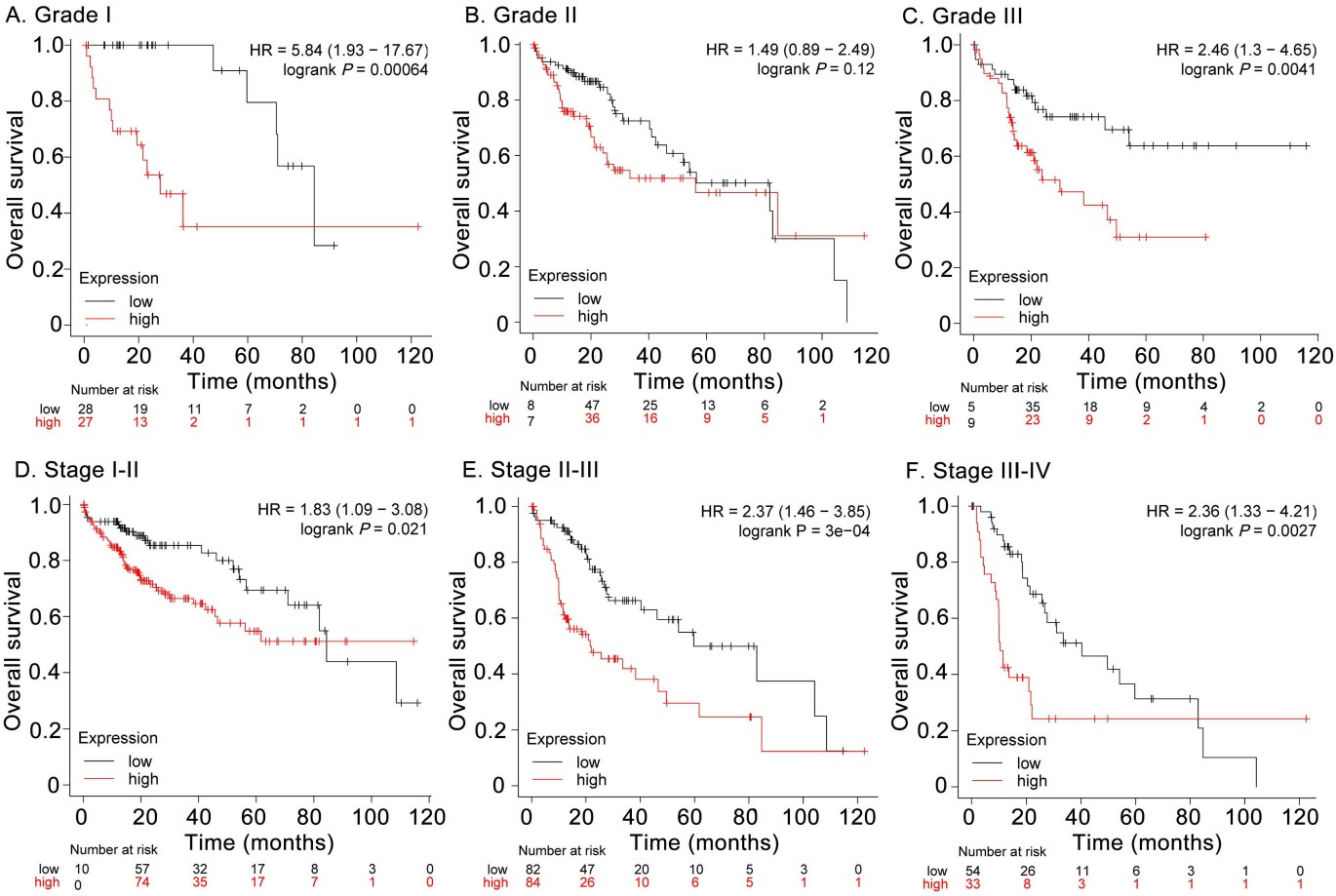
Overall survival of HCC patients grouped by* CENPF* median in different grades (A, B, C) and stages (D, E, F).

For subgroup survival analyses, upregulation of *CENPF* was significantly associated with poor OS in HCC patients with neoplasm histologic grade I and grade III (HR = 5.84, log rank* P* = 0.00064 and HR = 2.46, log rank *P* = 0.0041, respectively, Figures 6A and C), while no significant difference was found in HCC cases with grade II (HR = 1.49, log rank* P* > 0.05, Figure 6B). In addition, the overexpression of *CENPF* was closely related to poor OS in HCC patients with stage I-II (HR = 1.83, log rank *P* = 0.021, Figure 6D), stage II-III (HR = 2.37, log rank *P* = 3E-04, Figure 6E), and stage III-IV (HR = 2.36, log rank *P* = 0.0027, Figure 6F).

Besides, we conducted subgroup survival analysis in different population. As shown in Figure S1, high expression of *CENPF* was significantly associated with worse OS in HCC patients without hepatitis virus infection (HR =1.87, log rank *P* = 0.0073, Figure S1B). Overexpression of* CENPF* was significantly and negatively associated with OS both in men and women of HCC (HR = 1.93, log rank* P* = 0.0038 and HR = 2, log rank *P* = 0.017, respectively, Figures S1C and S1D). *CENPF* was a risk factor for OS in Asian patients with HCC (HR = 4.26, log rank *P*=7E-06, Figure S1F) and in HCC cases with alcohol consumption (HR = 1.97, log rank* P* = 0.0036, Figure S1G).

### Inhibition of *CENPF* expression affects the proliferation, migration, and apoptosis of HCC

**Figure 7:**
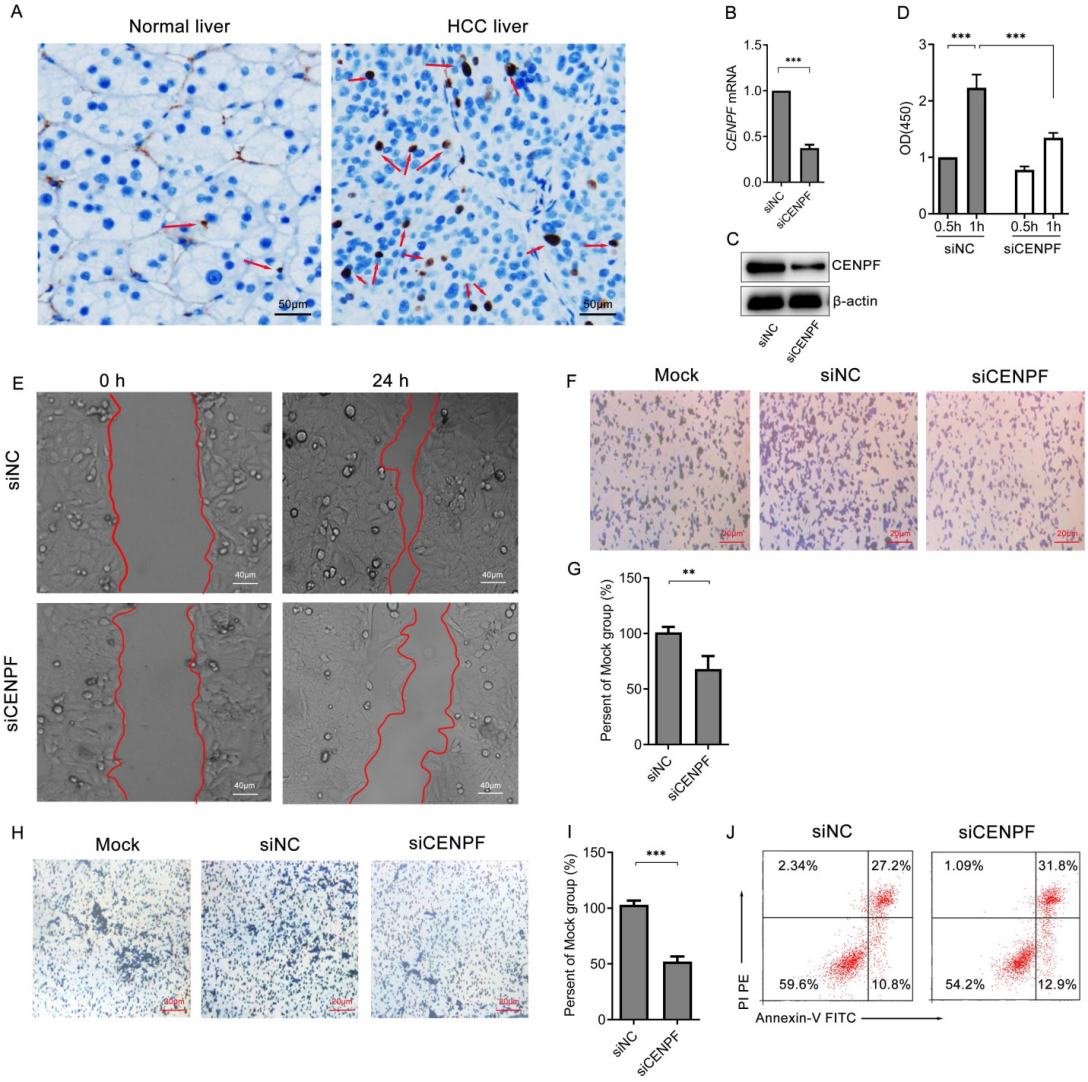
The expression of *CENPF* is involved in the cell proliferation and migration of HCC. (A) Immunohistochemistry of CENPF in human HCC samples; Blue represents hematoxylin (nucleus) and dark brown represents CENPF protein. The red arrows denote the expression of CENPF protein. (B) qRT-PCR and (C) Western blotting for CENPF expression in HepG2 cells after cells were transfected with homo-CENPF siRNA (siCENPF) or contorl siRNA (siNC) for 48 hours. β-actin molecular weight: 42 kDa, CENPF molecular weight: 330 kDa. (D) Cell Counting Kit-8 experiment for cell proliferation assay. (E) Wound healing assay for cell mobility investigation; The white line reflects the migration ability of HepG2 cells transfected with siCENPF or siNC for 24 hours. (F) Cell metastatic ability assay of HepG2 cells transfected with siCENPF or siNC for 24 hours. (G) Percentage of migratory cells compared to mock group. (H) Cell invasion assay of HepG2 cells transfected with siCENPF or siNC for 24 hours. (I) Percentage of invasive cells compared to mock group. (J) Flow cytometry analysis of apoptosis of cells using Annexin-V/PI kit.

Firstly, human HCC samples were used to confirm the expression of *CENPF*. According to the screening criteria for PPFE samples of HCC patients, 5 HCC tissues and 5 paired adjacent non-cancerous samples of PPFE were selected for IHC. As result, CENPF protein is over-expressed in human HCC tissues, compared with paired adjacent non-cancerous samples. (Figure 7A) According to The Human Protein Atlas database, CENPF was also high expressed in HepG2 cells. (Figure 2G) Thus, *CENPF* siRNAs (siCENPF) was constructed to investigate the role of *CENPF* in human HepG2 cells. The inhibition efficiency of siCENPF was more than 60% at RNA and protein levels for *CENPF*, compared with siNC. (Figures 7B-C) In CCK-8 cell proliferation experiment, down-regulation of *CENP*F significantly inhibited cell proliferation in human HepG2 cells at 1 hour (Figure 7D) and cell mobility at 24 hour (Figure 7E). Down-regulation of *CENPF* significantly inhibited cell migration (Figures 7F-G) and cell invasion (Figures 7H-I) in human HepG2 cells. Additionally, flow cytometry analysis showed that down-regulation of *CENPF* slightly promoted cell apoptosis in human HepG2 cells (27.2% vs 31.8%, Figure 7J).

### GSEA outlines underlying roles of *CENPF* in oncogenic signaling pathway

**Figure 8:**
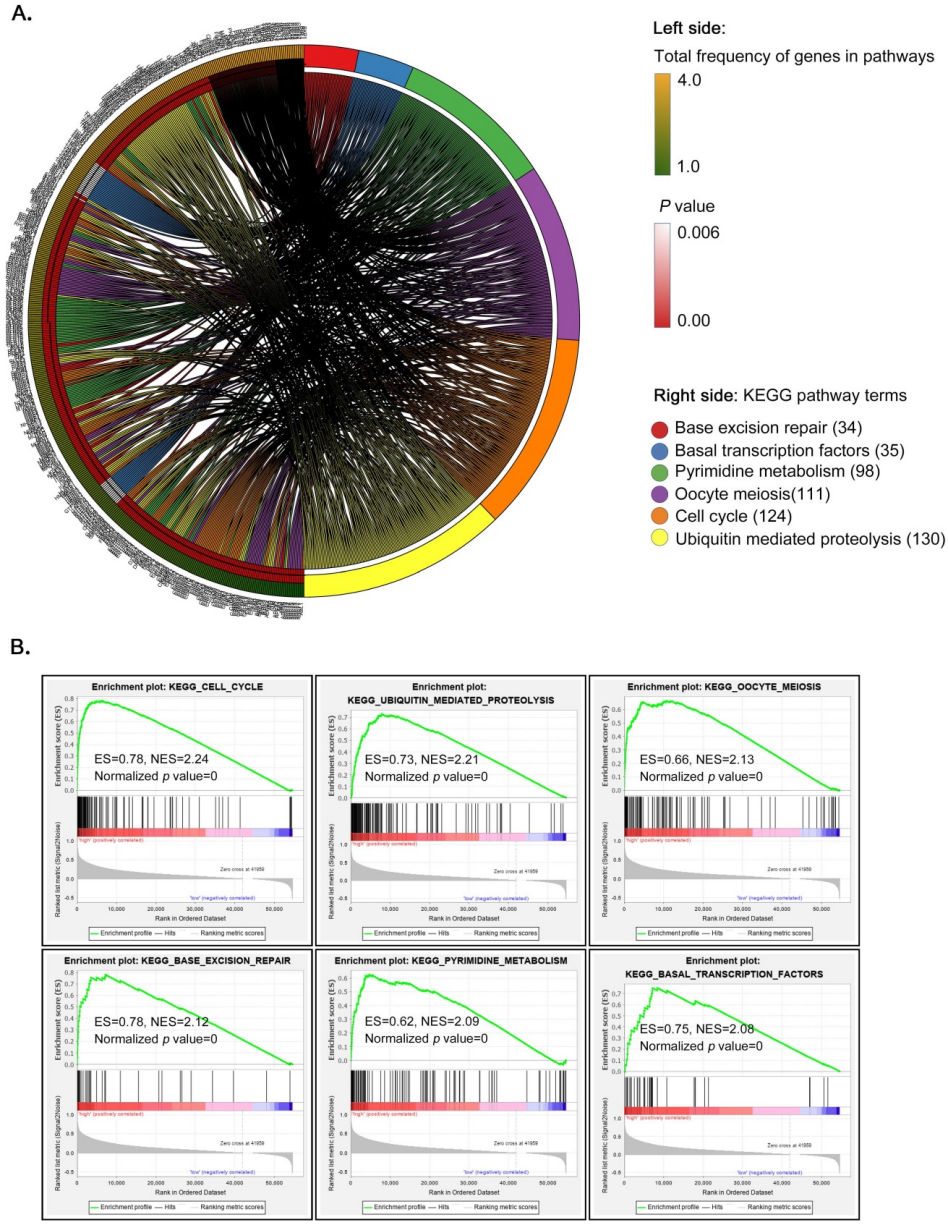
GSEA enrichment analysis of KEGG pathways in HCC samples with CENPF^high^ versus CENPF^low^. (A) Top 6 enrichment KEGG pathways and related genes; (B) Normalized enrichment scores and P-values of top 6 enrichment KEGG pathways.

To explore the potential molecular mechanisms by which *CENPF* alters tumor development and progress, GSEA enrichment analysis based on TCGA was utilized to analysis the gene expression profiles of CENPF^low^ and CENPF^high^ expression in HCC specimens. According to GSEA analysis of KEGG pathways, HCC with CENPF^high^ was mainly involved in cell cycle, ubiquitin mediated proteolysis, and oocyte meiosis, etc. (Figure 8A, and Table S2) The top 6 enrichment pathways and related genes were circular visualization and listed as Figure 8A. The normalized enrichment scores and* P*-values of GSEA analysis were figured out in Figure 8B.

### The *CENPF* may participate in cell cycle regulation and MAPK pathway in HCC

**Figure 9:**
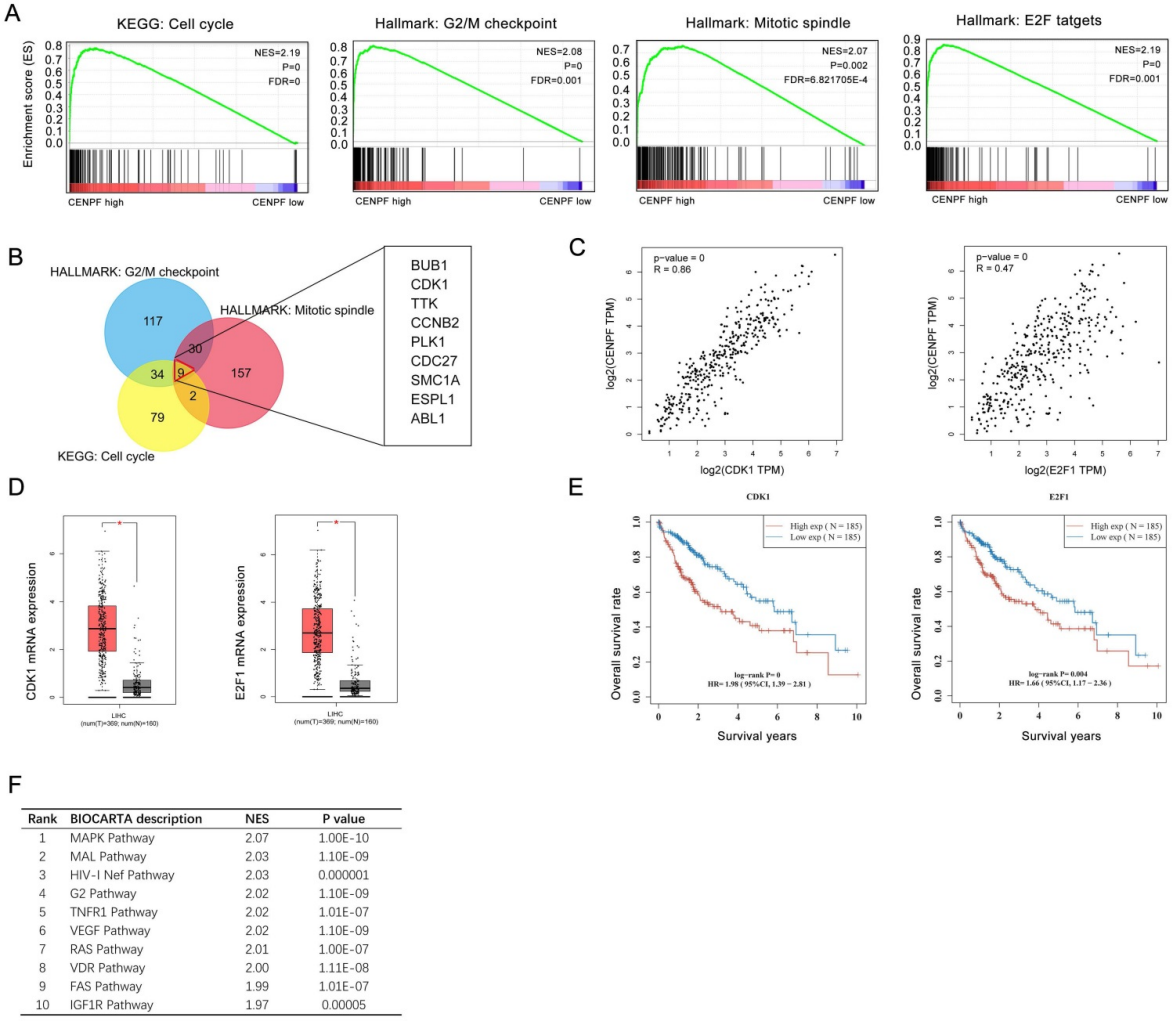
The *CENPF* may be involved in cell cycle and MAPK signaling pathway in HCC. (A) GSEA revealed that high CENPF expression was positively correlated with cell cycle pathway, G2/M checkpoint, mitotic spindle, and E2F targets. (B) Venn graph showed that BUB1, CDK1, TTK, CCNB2, PLK1, CDC27, SMC1A, ESPL1, and ABL1 were overlapped in the three groups. (C) Correlation of CDK1, E2F1 and* CENPF* expression in hepatocellular carcinoma. (D) Expression of CDK1 and E2F1 in hepatocellular carcinoma and normal liver tissues. (E) Prognostic analysis of CDK1 and E2F1 in hepatocellular carcinoma by GEPIA. (F) Top 10 GSEA enrichment analysis of Biocarta description.

Furthermore, integrated GSEA enrichment analyses of KEGG pathway and Hallmark description were carried out to declare that cell cycle, G2/M checkpoint, mitotic spindle, and E2F targets were identified as the significant signatures affected by *CENPF*, indicating that *CENPF* might affect cell cycle by interacting with G2/M checkpoint, E2F targets, and mitotic process. (Figure 9A, and Tables S2-S3) It was reported that E2F1, a member of E2Fs family, was an pivotal transcription factor of *CENPF* in NCI-60 cell line 53, and E2F1 played an important role in G2/M transition and cell cycle regulation^54^, ^55^. Thus, we focused on E2F1 in HCC. Venn graph analysis demonstrated that 9 genes (*BUB1, CDK1, TTK, CCNB2, PLK1, CDC27, SMC1A, ESPL1*, and *ABL1*) were co-expressed in these three groups. (Figure 9B) By screening the correlation, expression data and prognosis information of these nine genes and* E2F1*, we found that *CDK1* and *E2F1* were evidently upregulated in HCC (Figure 9D, all *P*<0.05) compared with normal tissues, and significantly positively correlated with the expression of *CENPF* (Figure 9C, all *P*<0.01). The overexpression of *CDK1* and *E2F1* also led to poor survival prognosis of HCC patients (Figure 9E, all *P*<0.01). Thus, CENPF-E2F1/CDK1 pathway mediating abnormal cell division in cell cycle might be a indispensable process in hepatocellular carcinoma. As listed in Figure 9F and Table S4, GSEA enrichment analysis of Biocarta description revealed that overexpression of *CENPF* was mostly involved in MAPK mediated inflammatory signaling pathway in the development and progress of HCC.

### Signature analysis of immune infiltration in HCC by CIBERSORTx

**Figure 10:**
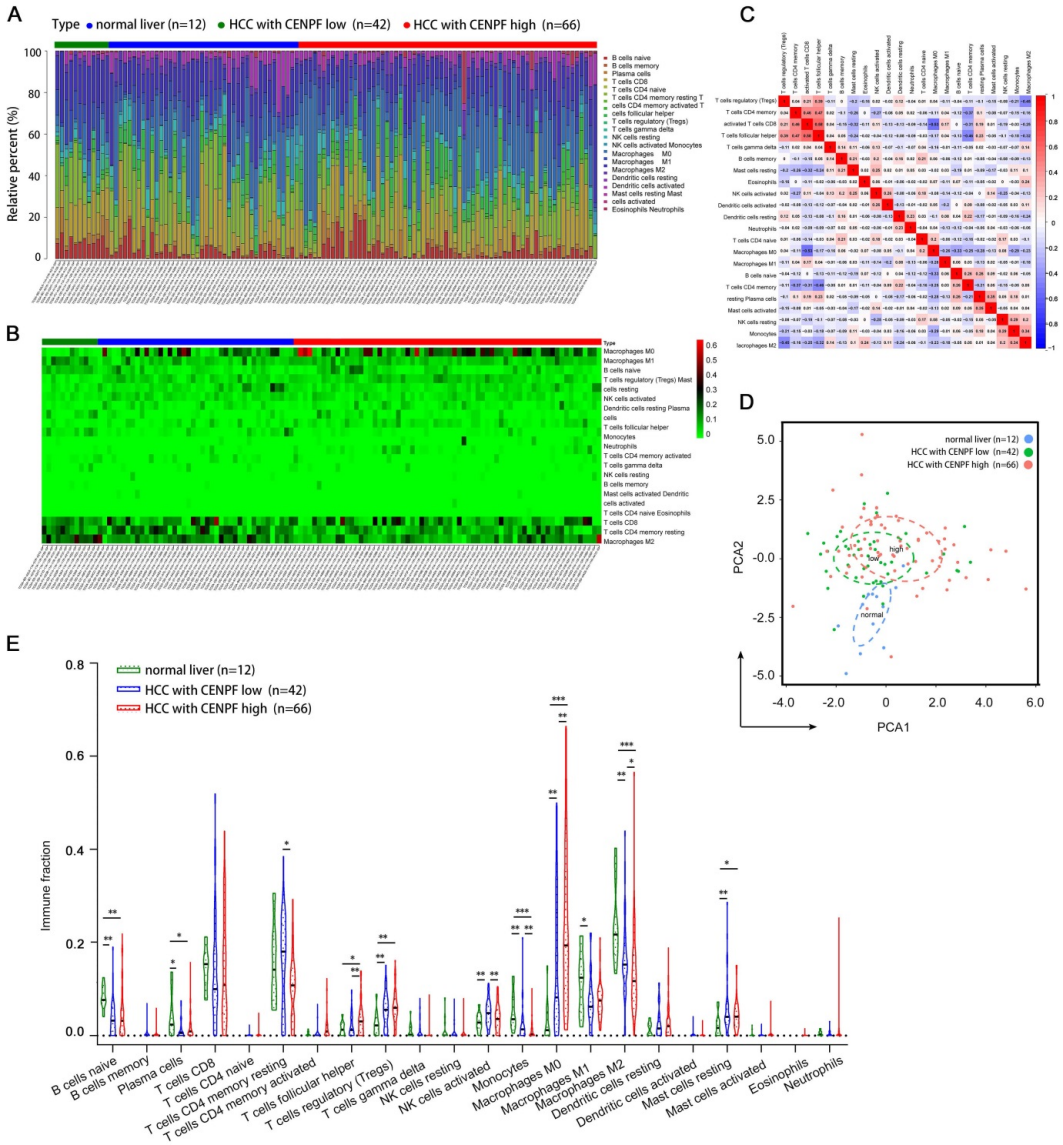
Immune cells infiltration analysis of three sub- groups including normal liver (n=12), CENPF^low^ (n=42) and CENPF^high^ (n=66) samples in HCC. (A) The relative percent of 22 types of immune cells in each sample. (B) The difference of immune cells infiltration for three groups visualized by heatmap. The correlation between the 22 immune cell populations(C) and PCA analysis (D), and immune fraction (E) in HCC samples.

The expression matrix from TCGA datasets contains 424 HCC tissues divided to three groups, including 50 normal liver samples, 187 CENPF^low^, and 187 CENPF^high^ HCC samples by median cutoffs, and normalized by 'Limma' packages. CIBERSORTx was applied to screen the 22 cell types potentially involved in the occurrence and development of HCC with low and high expression of *CENPF*. P<0.05 was considered as statistically significant. Then, the filtered information including 12 normal liver samples, 42 HCC samples with *CENPF* low expression and 66 HCC samples with* CENPF* high expression was considered for further analysis. The immune cell types of each sample in forms of relative percentage (Figure 10A), and expression level (Figure 10B) were shown as heatmaps. The correlation between the 22 immune cell populations in HCC samples was shown in the Figure 10C. The principal component analysis (PCA) revealed that there was significantly different in immune infiltration between normal liver and HCC samples, but not obvious difference was found between the HCC with low *CENPF* expression group and the HCC with high *CENPF* expression group. (Figure 10D) It might suggest that the immune heterogeneity of the CENPF^low^ and CENPF^high^ was not significant. Lastly, the immune enrichment fraction was significantly different in normal, 187 CENPF^low^, and 187 CENPF^high^ samples. (Figure 10E) As results in B cell population, the immune enrichment fraction of naïve B cells and plasma cells in HCC with CENPF^high^ group were much lower than that in HCC with CENPF^low^ group or normal tissues. Otherwise, the immune enrichment index of memory B cells in HCC and normal group were all very low, even could not be detected. In T cell population, T regulatory cells (T_reg_), and follicular helper T cells were manifestly enriched in HCC with CENPF^high^, compared with HCC with CENPF^low^ or normal ones. However, there was no difference for CD8 and CD4 T cells. In NK cell population, the immune enrichment score of activated NK cells in HCC with CENPF^high^ was slightly higher than that in HCC with CENPF^low^, but the difference was not significant compared with normal tissues. Moreover, the immune enrichment score of monocytes, macrophages M2 in HCC with CENPF^high^ than that in HCC with CENPF^low^ or normal tissues. But for macrophages M0, the immune enrichment score gradually increased in the normal, CENPF^low^, and CENPF^high^ groups. In granulocytes group, the immune enrichment score of resting mast cells in HCC with CENPF^high^ or CENPF^low^ were higher than that in normal tissues. But for activated mast cells and eosinophils, the scores were all very low, even could not be detected.

**Figure 11:**
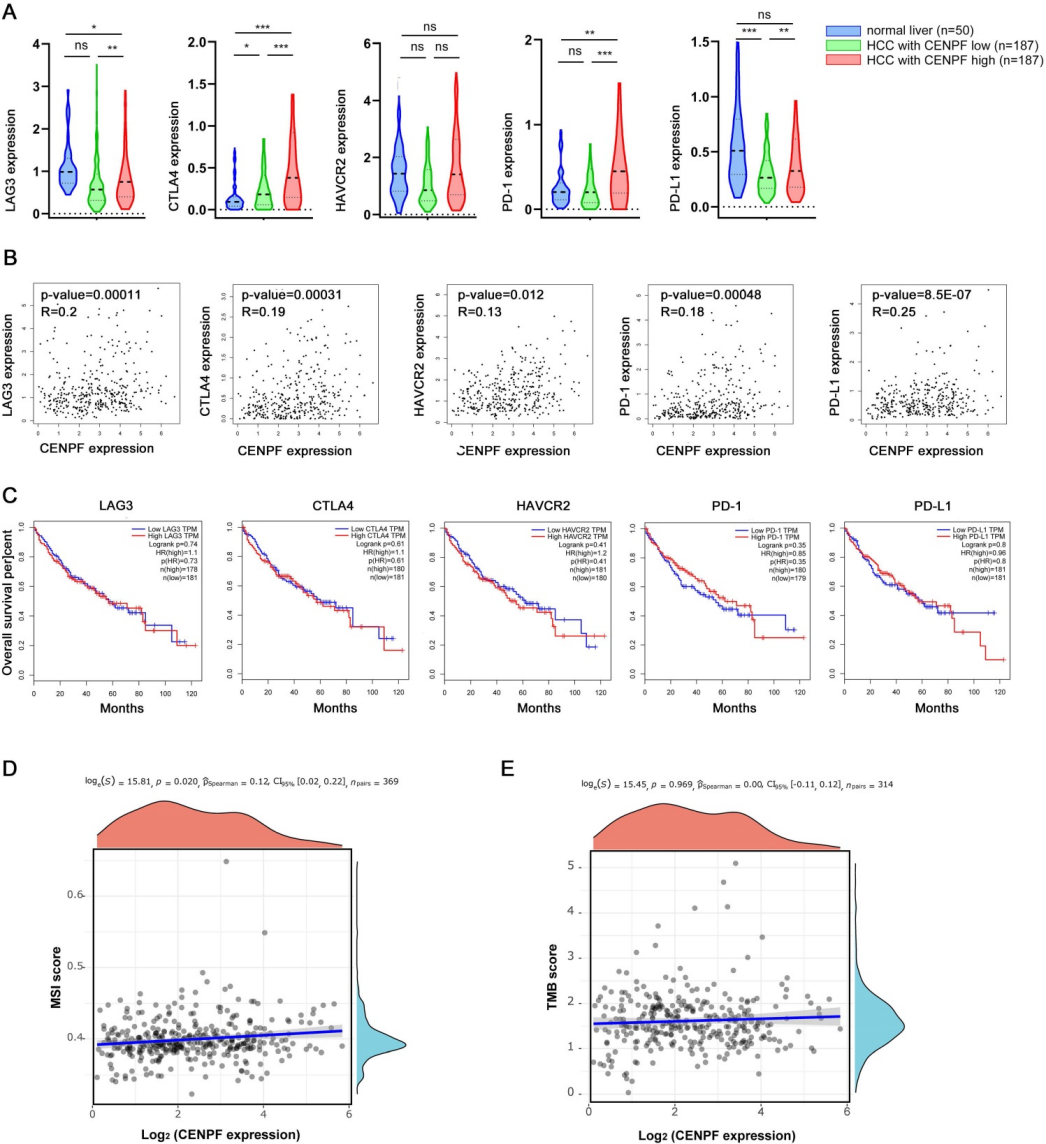
Objective to screen the expression, correlation and prognosis of *CENPF* with these five genes (LAG3, CTLA4, HAVCR2, PD-1, and PD-L1), MSI and TMB in hepatocellular carcinoma. (A) The expression of these five genes in CENPF^high^, CENPF^low^ , and normal liver tissues. (B) The correlation between CENPF and five genes in HCC. (C) Survival analysis of the five genes in HCC. Correlation analysis of *CENPF* expression and MSI(D) and TMB(E) in HCC. The horizontal axis in the figure represents the expression distribution of the gene, and the ordinate is the expression distribution of the TMB/MSI score. The density curve on the right represents the distribution trend of the TMB/MSI score; the upper density curve represents the distribution trend of the gene; the top side. The value represents the correlation p value, correlation coefficient and correlation calculation method.

In addition, the mRNA levels of* LAG3*, *CTLA4*, *HAVCR2, PD-1*, and *PD-L1* in HCC samples, which were closely related to tumor immunosuppression or tolerance, were shown in Figure 11. The expression of *LAG3*, *CTLA4*, *PD-1*, and* PD-L1* were upregulated in HCC with CENPF^high^, compared to HCC with CENPF^low^. (Figure 11A) Moreover, the correlation between the expression of these five genes and *CENPF* were further explored in HCC patients. The expression of *LAG3*,* CTLA4*, *HAVCR2*, *PD-1*, and *PD-L1* were not markedly positive correlated with *CENPF* (Figure 11B, all R<0.75), and there was no significant correlation between the expression of these five genes and the overall survival rate in HCC patients (Figure 11C, all *P*>0.05). Lastly, the correlation between *CENPF* expression and MSI or TMB were also screened. It showed that the expression of *CENPF* was not significantly related to MSI or TMB in HCC (Figures 11D-E, all the value of Spearman's correlation coefficient, |ρ _Spearman_ |, is close to 0).

## Discussion

Numerous studies have manifested that *CENPF* is not only involved in cell proliferation but also in tumorigenesis 26-28. In the past few years, evidence has accumulated that abnormal expression or activation of *CENPF* is a common phenomenon in malignant tumors, and there is a significant correlation between* CENPF* and tumorigenesis or progression in cancer patients, including HCC 27, 28, prostate cancer, breast cancer 29, and other tumors 30, which has been partially confirmed. Aytes A, et al. reported that *CENPF* has been identified as a major co-regulator of prostate cancer and a poor prognostic indicator of survival and metastasis 31. However, the patterns of expression and the exact roles of *CENPF* in HCC patients are not yet well known, and the molecular mechanism and the functions of *CENPF* remain undefined. The purpose of this study was to systematically investigate the expression alteration, prognostic values, correlation, and potential functions of *CENPF* in HCC.

Integration of multiple arrays has been viewed as a more reasonable method to improve detection capabilities and enhancing the reliability of results than single array analysis 56. In present study, we have gained insight into gene expression of *CENPF* through analyzing HCC datasets from GEO, TCGA and Oncomine. Moreover, protein expression of CENPF in HCC tissues and their associated normal tissues were also screened in The Human Protein Atlas by IHC staining. The staining intensity of CENPF antibody was mainly in HCC tissues and weakly positive in normal adjacent tissues. The information from GEO datasets indicated that *CENPF* was upregulated in HCC, including primary and metastasis with lung or adrenal gland tissue, compared with normal ones. But no significant difference was found between primary HCC and HCC with lung or adrenal gland metastasis tissues. Consistent with the results, *CENPF* was found to be significantly upregulated in cirrhosis, liver cell dysplasia and HCC tumors compared with normal livers. Additionally, Sung WK et al. reported that the expression of *CENPF* is significantly higher in HCC tissues than that in cirrhosis 57. Wurmbach E et al*.* reported that mRNA level of *CENPF* is significantly upregulated in HCC tissues than that in cirrhosis, in liver cell dysplasia and in normal livers. But no significant difference was found between cirrhosis or liver cell dysplasia and normal livers. 58 Based on TCGA datasets, uni-cox and mult-cox analysis was performed to figure out that the *CENPF* expression and pTNM stage might be independent prognostic factors in HCC patients. Hence, we assumed that *CENPF* may be emerging as a biomarker to distinguish HCC from normal livers, but further studies should be conducted to discriminate benign liver lesions from normal livers.

Then, Kaplan-Meier analyses of *CENPF* were performed to show that high expression levels of *CENPF* in tumor were significantly associated with the deterioration of OS and PFS in HCC patients. Overexpression of *CENPF* were associated with poor 1-year, 3-year and 5-year OS, and it seemed that hazard ratio (HR) of *CENPF* overexpression associated with OS was more obvious in the early years of HCC. In subgroup analyses, overall survival comparison in population of Asian, both male and female and without hepatitis virus infection were significantly associated with worse OS in HCC patients with *CENPF* overexpression (Figure S1). It indicated that this may provide a clue to the prognosis or treatment of HCC patients with high expression of* CENPF* and non-hepatitis virus infection. A series of publication reinforced our results. Dai Y et al*.* showed that upregulation of *CENPF* in HCC is positively associated with serum AFP, venous invasion, advanced differentiation stage and a shorter overall survival, and overexpression of *CENPF* was a risk factor for the prognosis of HCC. Inhibition of *CENPF* expression could reduce the ability of cell proliferation, migration, and tumor formation in nude mice, and block the cell cycle at G2/M checkpoint. 27 Kim H E et al*.* reported that gene amplification of *CENPF* is closely related to tumor formation and development, and it plays a role as a cancer-driver gene in human cancers 59. A recent study identified that overexpression of *CENPF* and/or Lymphoid-specific helicase (*LSH*) are correlated with shorter overall survival and higher cumulative recurrence rates in HCC patients. *LSH* may promote the development of HCC by transcriptional regulation of* CENPF* expression 28. Generally, our findings suggested that *CENPF* was overexpressed in HCC and might play a critical role in driving HCC tumorigenesis. Functionally, as a member of centromere protein family, *CENPF* might exert a pivotal role through regulating chromosome segregation during mitosis cell cycle at G2/M checkpoint.

For the purpose of verifying the above results, immunohistochemistry for the human samples was performed to confirm that the expression of *CENPF* was upregulated in HCC patients, compared with paired adjacent tissues. Furthermore, a down-regulation system of *CENPF,* siRNA, was constructed to probe the effect of *CENPF* on the function of hepatoma cells in the cell line of human HepG2 cells. The results asserted that down-regulation of *CENPF* inhibited the proliferation, migration, and invasion of HCC cells, and slight promoted the apoptosis of HCC cells (27.2% vs 31.8%, Figure 7J). It suggested that* CENPF* might play a crucial role in promoting the proliferation, migration, invasion, and inhibiting apoptosis of HCC cells during the progression or deterioration of HCC.

Then, the Gene-set enrichment analysis (GSEA) of KEGG pathway from TCGA datasets in current study pointed out that CENPF^high^ was mostly enriched in cell cycle pathway, closely related to the occurrence of a variety of tumors. Meanwhile, according to Hallmark description and Biocarta description, we found that *CENPF* was closely related to E2F1 and CDK1in the regulation of cell cycle, especially during G2 / M transition of mitosis. In addition, *CENPF* might also participate in MAPK signaling pathway in the development and progression of HCC. Therefore, our viewpoint threw light on the growing evidence regarding *CENPF* interactive genes and their associated signaling pathways, which might provide clues for the development of *CENPF*-mediated targeted therapy. However, further molecular mechanism, pharmaceutical and clinical studies are needed to confirm this prediction. Unfortunately, further experimental research for exploring the latent carcinogenic mechanism of *CENPF* in the occurrence and development of HCC could not be carried out by us. And, we did not have our own follow-up information of HCC patients.

As far as we know, HCC is tumor with primarily resistance to chemotherapeutic drugs 18. Although novel biomarkers and multiple molecular mechanisms of HCC have been studied, effective drugs for the treatment of HCC are still scarce 60. As one of hallmarks of cancer, immune microenvironment and immunotherapy has attracted much attention in the research of liver cancer in recent years. The presence of lymphocytic infiltration in tumors has been considered to be one of the prerequisites for immunotherapy 61. In this paper, immune cell infiltration analysis of HCC with *CENP*F high and low expression was constructed by CIBERSORTx to reveal that the immune cell types were significantly different in HCC samples with CENPF^high^, compared with normal liver samples, and HCC samples with CENPF^low^. As results, T regulatory cells (T_reg_), follicular helper T cells, macrophages M0, and resting mast cells were manifestly enriched in HCC with CENPF^high^, compared with CENPF^low^ or normal ones. While monocytes, macrophages M1 and M2, activated NK cells were downregulated in CENPF^high^ group, compared with CENPF^low^ group. The proportion B cells was low, and most of them were in the state of naïve B cells. The result declared that immunosuppressive microenvironment has played predominant roles in the occurrence and development of HCC with *CENPF* high expression, such as high immune infiltration score of T_reg_, resting mast cells, and down-regulation of monocytes and neutrophils.

A variety of immunosuppressive mechanisms have been found in tumor microenvironment, including *PD-1 / PD-L1*, *CTLA-4*, *PD-L1*, *LAG3*, etc. Thus, five genes closely related to immunotherapy were investigated in this study. As reported, *LAG-3* was confirmed to be involved in the proliferation of T cells *in vitro* and *in vivo*. Costimulatory molecules, including* LAG3*, *PD-1*, *CTLA4,* and *TIGIT*, are expressed on dysfunctional or depleted T cells in chronic viral infection and tumor 62. Tumor growth was inhibited in *LAG3* knockout mice 63. As results, *LAG-3, CTLA-4, PD-1,* and* PD-L1* were enriched in in HCC samples with CENPF^high^, compared with CENPF^low^ or normal groups, but no significant positive correlation was found between expression of *LAG3*,* CTLA4*, *HAVCR2*, *PD-1*, and *PD-L1* and the expression of* CENPF*. The OS of the five gene was not statistically significant in HCC patients. Therefore, HCC patients with CENPF^high^ group may not benefit from immunotherapy targeting these five genes.

The incidence of MSI is high in a variety of tumors, such as endometrial carcinoma, intestinal adenocarcinoma, gastric adenocarcinoma and so on. As a molecular marker of tumor cells, MSI-H plays an important role in predicting the antitumor effect of PD-1 / PD-L1 inhibitors 64, 65. Additionally, clinical studies have shown that TMB is closely related to the efficacy of existing PD-1 / PD-L1 inhibitors. Patients with high TMB are more likely to benefit from immunotherapy. TMB, as an independent factor of immune response to PD-L1, has a positive linear correlation with immune response, and its scope of application is not limited by pathological types 66. However, the expression of *CENPF* was not significantly related to MSI or TMB in HCC with the *CENPF* expression.

Collectively, we speculated that immunotherapy targeting these five target genes (especially including PD-1 / PD-L1, MSI, and TMB) has no obvious effect in patients with HCC with high *CENPF* expression. However, several immunosuppressive regents, including T_reg_, resting mast cells, monocytes, and neutrophils, might be viewed as potential therapeutic targets for the immunotherapy of HCC patients with *CENPF*^high^.

Eventually, based on current reports, a hypothesis was cautiously drawn that overexpression of *CENPF* contributed to unfavorable prognosis in HCC patients. Inhibition of *CENPF* expression might be conducive to alleviate the progression or treatment of HCC.

In summary, our study gave results that the mRNA and protein expression levels of *CENPF* were significantly upregulated in HCC. Aberrant expression of *CENPF* was associated with the clinical prognosis of HCC patients. The overexpression of *CENPF* was tightly connected to the mitosis of cancer cells and the occurrence and development of HCC. The results suggested that* CENPF* may serve as potential prognostic biomarkers and targets for HCC patients. Meanwhile, these results may be helpful for us to better understand the molecular mechanism of HCC and useful to develop tools for more accurate HCC prognosis and promote the development of *CENPF*-mediated therapeutic drugs for HCC. Evidently, further research on the basic molecular mechanism and clinical application of* CENPF* are still needed to improve the clinical value of *CENPF* as a prognostic indicator or therapeutic target for HCC.

## Supplementary Material

Supplementary figures and tables.

## Figures and Tables

**Table 1:** The sequences information of qRT-PCR primers and siRNAs.

Gene name	Primers (5'-3')	
***CENPF***	Forward: AGCACTGATCACCTGTTAGC	Reverse: ACCCACATACAAACAGAGATTG
***GAPDH***	Forward: CGGAGTCAACGGATTTGGTCGTAT	Reverse: AGCCTTCTCCATGGTGGTGAAGAC
**siCENPF**	Forward: GACCCAGAAACUAGCUUAUTT	Reverse: AUAAGCUAGUUUCUGGGUCTT
**siNC**	Forward: UUCUCCGAACGUGUCACGUTT	Reverse: ACGUGACACGUUCGGAGAATT

siCENPF: *CENPF* siRNA, siNC: negative control siRNA.

**Table 2:** Details of HCC studies and associated microarray datasets from GEO database.

GEO Series	Contributor(s)	Sample		Platform	Country
		Tumor	Normal		
GSE14520	Roessler S et al, 2009	225	220	GPL3921 [HT_HG-U133A] Affymetrix HT Human Genome U133A Array	USA
GSE60502	Kao KJ, 2014	18	18	GPL96 [HG-U133A] Affymetrix Human Genome U133A Array	China (Taiwan)
GSE40367	Roessler S, 2012	10	5	GPL570 [HG-U133_Plus_2] Affymetrix Human Genome U133 Plus 2.0 Array	USA
GSE84005	Tu X et al, 2016	38	38	GPL5175 [HuEx-1_0-st] Affymetrix Human Exon 1.0 ST Array	China
GSE112791	Kaoru Mogushi et al, 2018	183	15	GPL570 [HG-U133_Plus_2] Affymetrix Human Genome U133 Plus 2.0 Array	Japan
GSE76297	Xin Wei W, 2015	61	151	GPL17586 [HTA-2_0] Affymetrix Human Transcriptome Array 2.0	USA
GSE25097	Zhang C, 2010	268	243	GPL10687 Rosetta/Merck Human RSTA Affymetrix 1.0 microarray, Custom CDF	USA
GSE87630	Woo HG, 2016	64	30	GPL6947 Illumina HumanHT-12 V3.0 expression	South Korea

**Table 3:** Characteristics of HCC patients between CENPF^high^ and CENPF^low^ groups (median cutoffs).

Variables	*CENPF* expression level		*P* value	Variables	*CENPF* expression level		*P* value
	Low (n = 187)	High (n =187)			Low (n = 187)	High (n = 187)	
**Gender, male (%)**	145 (77.5)	131 (70.1)	0.106	**Pathological stage, n (%)**			0.032
**Age, median (IQR), years**	64 (15)	59 (17)	0.196	I	107 (57.2)	84 (44.9)	
BMI, kg/m^2^, n (%)			0.025	II	42 (22.5)	53 (28.3)	
<18.5	0	0		III	35 (18.7)	55 (29.4)	
18.5~24.99	36 (19.3)	18 (9.6)		IV	4 (2.1	1 (0.5)	
25~29.99	106 (56.7)	109 (58.2)		NA	16 (8.6)	13 (7.0)	
>30	46 (24.6)	58 (31.0)		**Vascular invasion, n (%)**			0.072
NA	17 (9.1)	20 (10.7)		Macro	7 (3.7)	11 (5.9)	
**Tumor status, n (%)**			0.059	Micro	54 (28.9)	51 (27.3)	
With tumor	54 (29.9)	69 (36.9)		None	122 (65.2)	106 (56.7)	
Tumor free	139 (74.3)	118 (63.1)		NA	21 (11.2)	38 (20.3)	
NA	11 (5.9)	19 (10.2)		**Child-pugh classification, n (%)**			0.082
**Family history of cancer, n (%)**			0.036	A	129 (68.9)	112 (59.8)	
No	100 (53.5)	127 (67.9)		B	13 (6.9)	9 (4.8)	
Yes	70 (37.4)	52 (27.8)		C	1 (0.5)	0 (0)	
NA	34 (18.2)	27 (9.7)		NA	61 (32.6)	85 (45.5)	
Hepatocarcinoma risk factors*, n (%)			0.63	**New tumor event after initial treatment, n (%)**	90 (48.1)	112 (59.8)	0.038
Hepatitis virus infection	57 (30.9)	65 (34.8)		**Ishak fibrosis status, n (%)**			0.084
Alcohol consumption	75 (40.1)	60 (32.1)		No fibrosis	52 (27.8)	31 (16.6)	
Non-alcoholic fatty liver disease	8 (4.3)	8 (4.3)		Portal fibrosis	14 (6.8)	18 (8.8)	
No risk factors	46 (24.6)	52 (27.8)		Fibrous speta	15 (8.0)	15 (8.0)	
Other	9 (4.8)	12 (6.4)		Nodular Formation/Incomplete Cirrhosis	7 (3.7)	3 (1.6)	
NA	9 (4.8)	11 (5.9)		Cirrhosis	40 (21.4)	44 (23.5)	
**Neoplasm histologic grade, n (%)**			<0.01	NA	76 (40.6)	95 (50.8)	
Grade 1	44 (23.5)	18 (9.6)		**Hepatic inflammation, n (%)**			0.029
Grade 2	113 (60.4)	86 (45.9)		None	74 (39.6)	55 (29.4)	
Grade 3	40 (21.4)	90 (48.1)		Mild	53 (28.3)	54 (28.8)	
Grade 4	4 (2.1)	8 (4.1)		Severe	13 (6.9)	7 (3.7	
NA	3 (1.6)	4 (2.1)		NA	64 (34.2)	90 (48.1)	
**Years to last follow up, median (IQR), years**	0.9 (1.81)	0.73 (2.29)	0.06				

IQR, interquartile range; BMI, body mass index; AFP, alpha-fetoprotein; NA, not available. * Sum of all risk factors compared with no risk factors.
