# Supplementary material for: Centromere Protein F (CENPF) Serves as a Potential Prognostic Biomarker and Target for Human Hepatocellular Carcinoma
Source: J Cancer. 2021 Mar 15;12(10):2933–51. doi: 10.7150/jca.52187 (PMC8040902; doi:10.7150/jca.52187)
Supplement: Supplementary file 1 — Supplementary figures and tables. [file jcav12p2933s1.zip › Figure S1.docx]

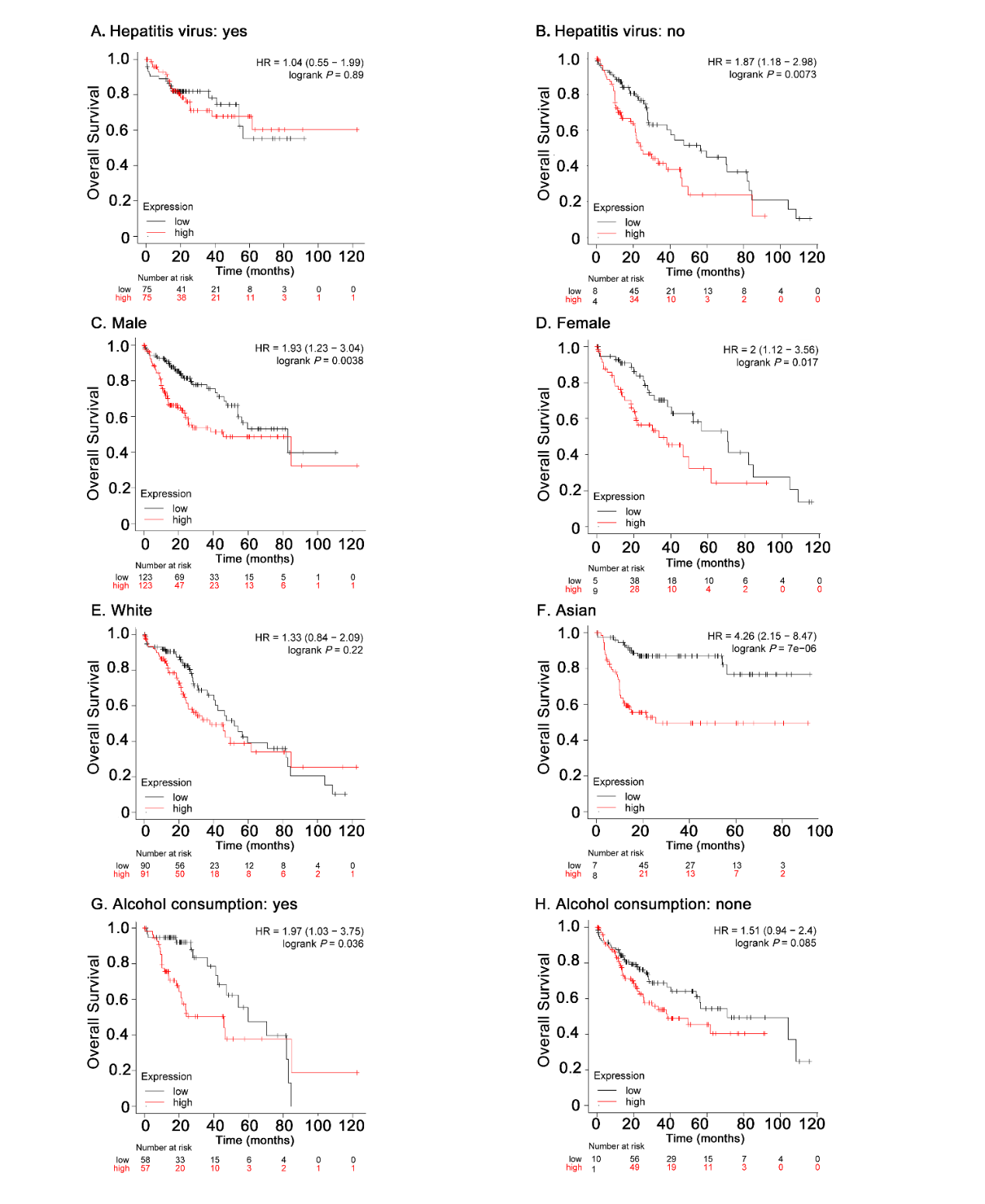


**Figure S1.** Subgroup analyses of overall survival comparison in different population, including hepatitis virus infection status (A, B), gender (C, D), race (E, F) and alcohol consumption (G, H)] with *CENPF* median cutoffs in HCC patients in Kapan-Meier Plotter.
